# Impact of demonstration in a realistic simulation environment as a postoperative education in patients’ experience

**DOI:** 10.31744/einstein_journal/2020AO4831

**Published:** 2020-03-13

**Authors:** Luciana Gardin Barbosa, Cinthya da Silva Frazão

**Affiliations:** 1 Associação de Assistência à Criança Deficiente São PauloSP Brazil Associação de Assistência à Criança Deficiente , São Paulo , SP , Brazil .

**Keywords:** Health education, Orthopedic procedures, Patient satisfaction, Simulation training

## Abstract

**Objective:**

To evaluate the impact of training in the Practical Life Room on patients experience during hospitalization.

**Methods:**

Subjects submitted to orthopedic surgeries were randomized to two groups (Control and Intervention) in the postoperative period. The Control Group received only the printed guidelines regarding the postoperative period, and the Intervention Group received the printed guidelines and a demonstration and training session with a physical therapist, in an environment created to simulate a house and its rooms (living room, bedroom, kitchen, laundry and bathroom). The participants of both groups answered the questionnaire Hospital Consumer Assessment of Healthcare Providers and Systems on the day of discharge.

**Results:**

Sixty-eight subjects were included in the study, 30 (44.1%) in the Control Group and 38 (55.9%) in the Intervention Group. The Hospital Consumer Assessment of Healthcare Providers and Systems questionnaire score showed no significant difference between the groups (p=0.496).

**Conclusion:**

There was no influence of the proposed intervention on the results of the Hospital Consumer Assessment of Healthcare Providers and Systems questionnaire, perhaps because of the limitation of the instrument or due to the fact it was employed when patients were still hospitalized. However, by reports from patients in the Intervention Group about felling better prepared and safer for performing daily activities, it is believed that patient education approaches through demonstration should be included as part of the process to prepare for discharge, whenever possible.

## INTRODUCTION

Major orthopedic surgeries, such as the correction of scoliosis, herniated discs and knee and hip arthroplasties, may result in limitations when it comes to activities of daily living, like showering, dressing, walking or cooking, when the patient is discharged. ^( 1 )^ Studies have suggested major surgeries can lead to temporary kinesiophobia, because the patient is afraid of performing movements or chores that might hinder their surgery. ^( 2 , 3 )^ During the first days after surgery, patients may partially or completely lose their independence, especially in the case of elderly individuals. ^( 4 - 6 )^

The literature recommends that discharge conditions should include, in addition to pain control, adequate movement range, muscle strength and the minimum required ability to move and walk – *i.e* . ensure that preoperative abilities remain unaltered or be reduced as little as possible after surgery. ^( 7 , 8 )^ In-hospital rehabilitation programs that include educating the patient through verbal or written guidelines, demonstrations or training for daily activities may help with quicker recoveries and reduce costs of readmissions, due to complications related to immobility or domestic accidents, such as falls. ^( 5 )^

Care teams use printed material to educate patients regarding the permitted activities, actions that require specific caution and those to be avoided after discharge according to the treatment given during the hospital stay. Strategies that anticipate possible difficulties in a practical manner and that include monitoring provide more safety during the postoperative period when the patient returns home. ^( 5 )^

The Hospital Consumer Assessment of Healthcare Providers and Systems (HCAHPS), ^( 9 )^ a validated questionnaire that measures patient satisfaction with the services provided during hospital stay, has been used as an evaluation tool by patients and family members regarding in-hospital services. It comprises 32 questions divided into the following domains: communication with doctors (doctors’ respect, ability to listen to patients’ requests, and communication skills); communication with nurses (nurses’ respect, ability to listen to patients’ requests, and communication skills); responsiveness of hospital staff (response to calls and requests to use the bathroom); hospital environment (cleanliness and quietness); pain management (staff’s ability to decrease patients’ physical pain); communication about medicines (explanations about medications to patients); discharge information (preparing the patient to leave the hospital); meal services (quality of food and politeness of meal delivery staff); general evaluation of the hospital (from 1 to 10).

In December 2017, the hospital of *Associação de Assistência à Criança Deficiente* (AACD) [Association for Care of Disabled Children] launched its Practical Life Room, which simulates the rooms in a house. In this space, patients rely on the guidance of a physical therapist who provides demonstrations and helps patients to simulate activities of daily living, such as lying down and getting up from bed, using the toilet, showering, using the computer on a desk, or even preparing a quick meal. The goal is to anticipate the difficulties that may arise after hospital discharge, explain to the patient how to overcome them and keep the patient active within the safety limits of their recovery. The patients are taken to this space as soon as their conditions allow it, *i.e* ., when there is no debilitating pain, symptoms or malaise of any nature.

Thus, it is believed that, in addition to explanations and informative booklets about the postoperative period, the opportunity to simulate daily activities can contribute to better patient recovery and reduce the risk of complications.

## OBJECTIVE

To evaluate the impact of training inpatients in the Practical Life Room.

## METHODS

The study included all patients who were at the hospital for postoperative care from orthopedic surgeries for cervical and lumbar disc herniation, correction of idiopathic scoliosis, and total knee and hip arthroplasty. Individuals who presented cognitive, clinical or motor alterations which limited their participation in this intervention were excluded from the study.

**Figure 1:**
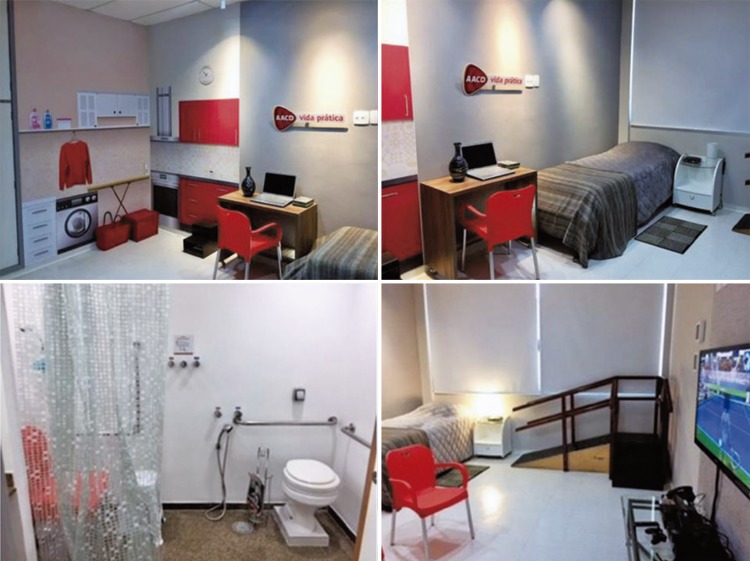
Practical Life Room

Initially, the hospital beds were randomized through an online randomization software into two groups (Control and Intervention). ^( 10 )^ Then, the patients were approached according to the bed they were assigned to. The Control Group received printed orientations that had been already used by the hospital (Annex 1), and the Intervention Group received the printed orientations in addition to a session of demonstrations and training in the Practical Life Room (Annex 2), which was focused on risk prevention and orientations to keep their functionality during the postoperative period. Figures 1 and 3 show the Practical Life Room and its areas.

**Figure 2:**
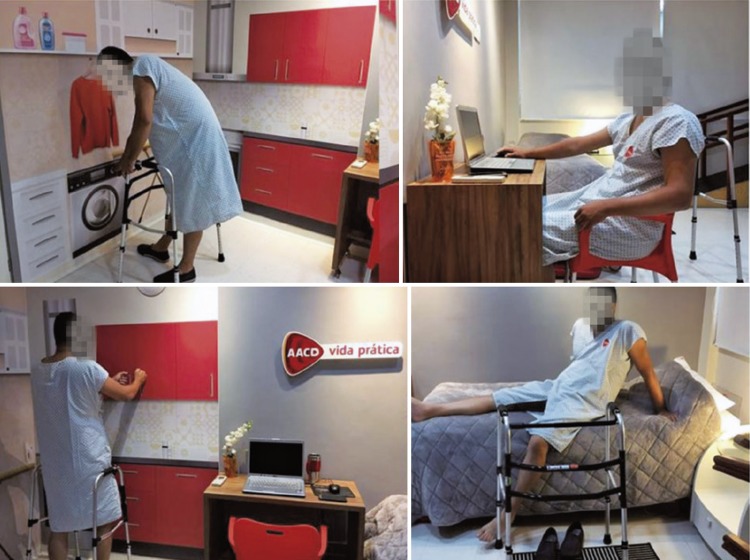
Training and demonstration to the patient in the Practical Life Room (laundry room, office, kitchen and bedroom)

**Figure 3:**
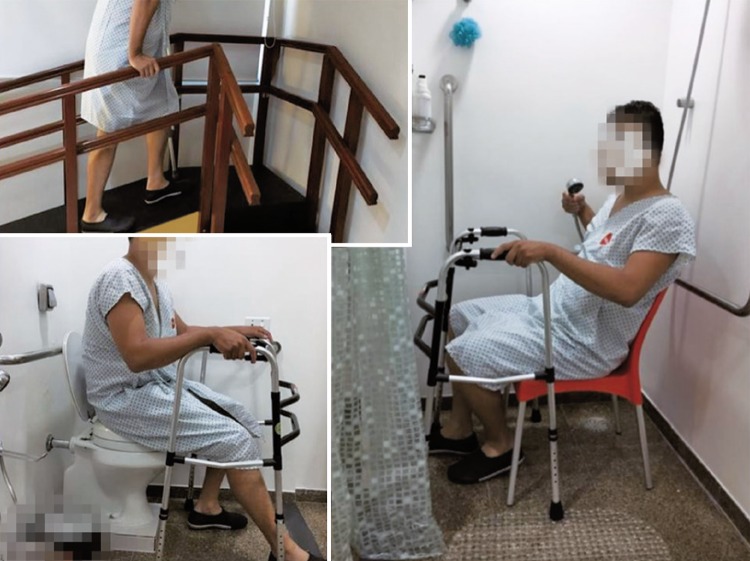
Training and demonstration to the patient in the Practical Life Room (ramps, stairs, toilet and shower)

The HCAHPS questionnaire was given to the patients included in the study on their discharge day. The individuals answered the questionnaire and handed it to a researcher who was blind to the randomization. The patients in the Control Group had the opportunity to go through the Practical Life Room in the first week after hospital discharge.

All variables were evaluated with descriptive statistics. The response variables, relative to the HCAHPS score, were tested in two ways: general HCAHPS, with all the obtained responses, and discharge HCAHPS, with only the questions related to the preparation for patient discharge (questions 18, 19, 20) to check the adherence to the normal distribution. After that, the Mann-Whitney and the Kruskal-Wallis tests were used to compare the scores between the groups, sexes and diagnoses. Quantitative variables were compared between the groups through the χ ^2^ test. The confidence intervals of the frequencies were obtained through bootstrapping.

This is a randomized, blind, controlled clinical trial approved by the Research Ethics Committee of *Hospital e Centro de Reabilitação da Associação de Assistência à Criança Deficiente* (CAAE: 88131418.0.0000.0085), under protocol number 2.792.735.

## RESULTS

Of the 71 individuals who began the study, 3 were excluded for not having answered the questionnaire in full (1 from the Control Group and 2 from the Intervention Group) Of the 68 individuals analyzed, 30 (44.1%) were in the Control Group and 38 (55.9%) were in the Intervention Group. The variable general HCAHPS presented normal distribution and the discharge HCAHPS did not (p<0.001); therefore, non-parametric tests were used.

The variable sex presented a difference between the groups (p=0.027). In the Intervention Group, male patients represented 23.7% of participants *versus* 50% in the Control Group. There was a homogenous distribution of diagnoses between the groups (p=0.838) and between the sexes (p=0.316). The results from the descriptive statistics of the qualitative variables, separated by group, are shown in table 1 .

**Table 1:** Qualitative variables of the study for the Control and Intervention Groups

	Control Group		Intervention Group	
	n (%)	95%CI	n (%)	95%CI
Sex				
Female	15 (50.0)	32.4-66.7	29 (76.3)	62.9-88.2
Male	15 (50.0)	32.4-66.7	9 (23.7)	11.8-37.1
Diagnosis				
Idiopathic scoliosis	3 (10.0)	0.0-22.6	8 (21.1)	8.8-35.3
Cervical disc herniation	7 (23.3)	9.7-40.0	9 (23.7)	11.1-38.1
Lumbar disc herniation	14 (46.7)	29.0-64.0	16 (42.1)	26.5-57.9
Knee arthroplasty	2 (6.7)	0.0-17.4	1 (2.6)	0.0-9.1
Hip arthroplasty	3 (10.0)	0.0-22.6	3 (7.9)	0.0-17.5
Knee prosthesis	1 (3.3)	0.0-11.1	1 (2.6)	0.0-9.1

The quantitative variables are shown in table 2 , where we can see that mean and median values were similar.

**Table 2:** 95%CI: 95% confidence interval. . Quantitative variables of the study for the Control and Intervention Groups

	Control Group		Intervention Group	
	Mean±SD (median)	P25-P75	Mean±SD (median)	P25-P75
Age	46.1±13.6 (46.5)	39.0-53	43.8±18.9 (44.5)	37-52
BMI	28.8±6.0 (27.9)	25.6-31.9	29.3±18.7 (27.1)	22.4-29.7
General HCAHPS score	79.5±6.9 (79)	75-86	80.8±6.0 (81)	76-86
Discharge HCAHPS score	3.6±0.9 (3)	3-4	3.5±1.0 (3)	3-4

SD: standard deviation; BMI: body mass index; HCAHPS: Hospital Consumer Assessment of Healthcare Providers and Systems.

The evaluation of the general and discharge HCAHPS scores showed similar distribution between the Intervention and Control Groups (p=0.496 and p=0.400, respectively).

**Figure 4:**
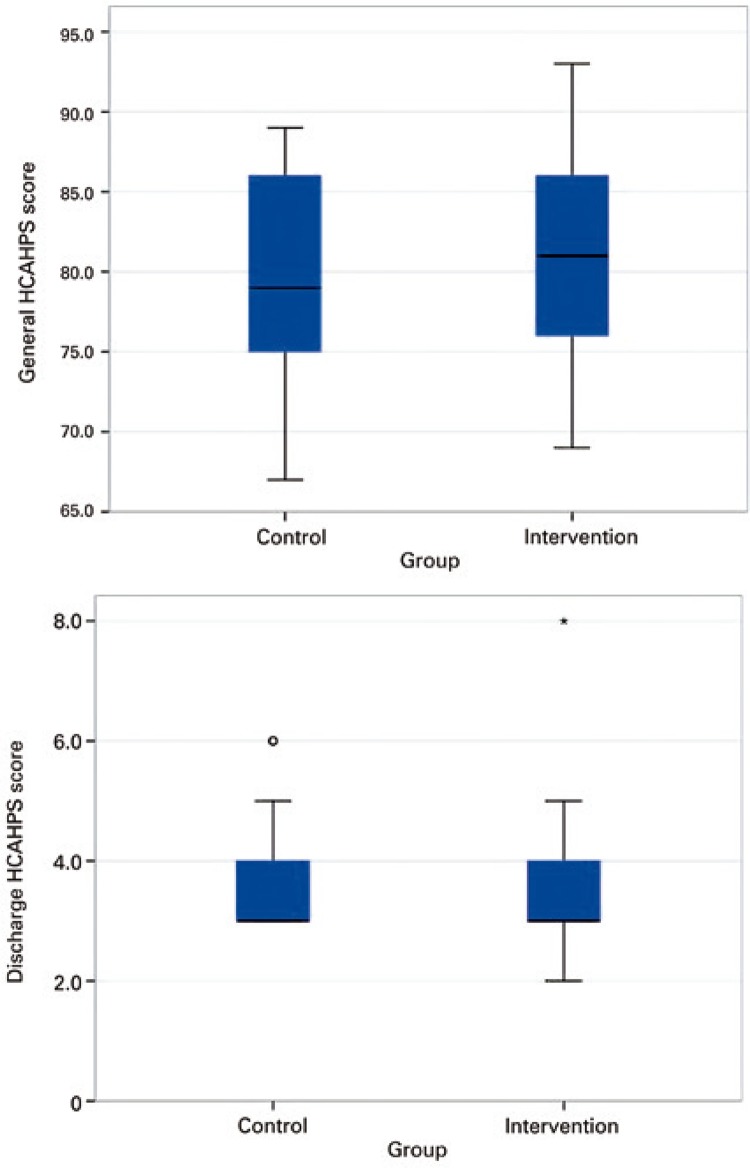
Boxplot to compare the groups in relation to the general and discharge Hospital Consumer Assessment of Healthcare Providers and Systems scores HCAHPS: Hospital Consumer Assessment of Healthcare Providers and Systems.

Visually, the boxplot graphs ( Figure 4 ) show the overlay of two groups, in agreement with the results of the statistical test.

When we compared the individual HCAHPS questions between the groups, only question 11 showed a difference between the groups (p=0.044); the other questions did not show statistical significance (between p=0.116 and p=1.0).

There was no difference in the general and discharge HCAHPS or in individual questions (significance between p=0.119 and p=1.0) between the sexes.

There was a difference in the evaluation of the discharge HCAHPS between the diagnoses. Patients with total hip arthroplasty presented a higher score than those with total knee arthroplasty, cervical and lumbar disc herniation (p=0.024, p=0.027, and p=0.05, respectively). However, there was no difference between the Control and Intervention Groups within each diagnosis. This difference in the discharge HCAHPS score was due to a better performance by patients with a diagnosis of total hip arthroplasty on question 19 (p=0.001).

## DISCUSSION

Patients with degenerative pathologies of the musculoskeletal system face daily limitations due to symptoms like pain, limitations of joint mobility, and movement disfunction, which can reduce their social and physical activities. ^( 11 - 13 )^ Page et al., in addition to those limitations, describe an impact on sleep and emotional suffering among the limitations referred by patients with degenerative pathologies of the shoulder. ^( 14 )^

The main goal of surgery is to reduce these limitations, and rehabilitation should provide the recovery of motor and respiratory functions as quickly as possible. The patient’s involvement in this process is paramount for positive outcomes.

McCormick et al. say there is a tendency to erroneously evaluate patients’ needs when we use traditional methods based solely on the experience of healthcare professionals, because these methods may not describe the perception of the patient about their health status. ^( 15 )^

When the care is focused on the patient and their families, when their preferences and beliefs are respected, and their needs are met to ensure the continuity of care, the results and experiences during hospital stay are better, and contribute to an improvement of their mental health status and the length of hospital stay is reduced. ^( 16 )^ Goldfarb et al., highlighted that this type of approach offers preventive care and reduces the number of undesirable treatments, thus improving the quality of care and leading to reports of satisfaction with the provided services. ^( 17 )^

Patient care during the transition from the hospital to the patient’s home must be personalized and prioritize quality to lower the risks in the post-discharge period. These risks are heightened if there is not an adequate process of orientation and education to the patient while he is still in the hospital. These orientation strategies are directly linked to a better adherence by the patient to the treatment, be it among pediatric, adult or geriatric patients. Written guidelines are often underexplained and generic. ^( 18 )^

In our study population, the most prevalent surgery was repair of herniated lumbar disc, since our service is a reference for spinal surgery. There was also a predominance of middle-age, obese, and female patients. Although there was a difference in the frequency of sex between the groups, this did not impact the results because there was no difference in the final score in relation to sex.

The results do not show significant differences between the Intervention and Control groups regarding the general and discharge HCAHPS scores. This result may be because there was an insufficient number of patients to demonstrate the effect of the intervention or because the measurement tool was not adequate.

Although the study’s measurements were not done objectively, it is worth mentioning that, during clinical practice, most family members and caretakers did not feel confident and properly prepared to care for the patients after discharge. After the interventions, be it with written guidelines or practical sessions in the Practical Life Room, family members and caretakers reported more confidence and satisfaction with care provided, which corroborates the importance of the structure of this process in healthcare organizations.

A recent study reported that using visual aids establishes more involvement and better understanding by patients during discharge instructions. ^( 18 )^ However, our study was inconclusive in the evaluation of the resources employed at the AACD hospital regarding patient experience, and we suggest further studies be conducted.

The moment when the questionnaire was applied may also have been a study limitation, because the HCAHPS methodology recommends it be answered after a few days at home. Due to some limitations intrinsic to our research team, the questionnaire had to be applied at the moment the patient was discharged, and we believe the perception of the benefits of the intervention cannot be noticed at that stage.

One finding of the study was the difference in the discharge HCAHPS evaluation between the diagnoses. The patients with total hip arthroplasty presented a higher score than those with total knee arthroplasty, cervical and lumbar disc herniation. The difference was related to the scores of question 19, which asks: “during this hospital stay, did physicians, nursing staff or other hospital staff talk with you about whether you would have the help you needed when you left the hospital?”, and the “no” answer occurred mainly in the group of patients undergoing hip arthroplasty. This may denote a difficulty to measure the need for help in the postoperative period for this specific population. Moreover, even though this result was not one of the goals of this study, it brought us important information which will be taken into account when we construct our patient education processes.

Thus, while the obtained results might not express significant differences and do not correspond to a greater satisfaction reported by the patients, we believe that the more different approaches a hospital can implement in the patient/caretaker education process during hospital stay, the better the continuity of care will be at home. Maybe this could bring a positive impact to postoperative recovery. However, to accurately measure this effect, a specific study must be conducted.

## CONCLUSION

Our study was not able to demonstrate the influence of training in the Practical Life Room on patients’ satisfaction with the services provided during their hospital stay, measured by the questionnaire Hospital Consumer Assessment of Healthcare Providers and Systems upon discharge. An additional study in which the questionnaire is applied in the first week after discharge, or one that uses a tool that is more sensitive to this intervention is necessary. However, reports from patients who underwent the intervention showed they were more prepared and confident to execute activities of daily living. We believe that the strategy of educating the patients through demonstrations should be included in the process of discharge preparation whenever possible.

## Annex I

**Table:**

Postoperative orientations given to patients (example for total knee prosthesis)
**Physical therapy orientations for total knee arthroplasty**
This manual was written by the physical therapy team of the hospital to guide the postoperative care.
By following the guidelines below, you will be contributing to the success of the surgery and will be able to more quickly resume your daily activities, and recover your independence.
Here are some orientations you were given during your hospital stay, which must be followed at home until you see your physician again:
Definition
Total knee arthroplasty is the replacement of the original damaged joint for a new joint called knee prosthesis. It is made of special materials that try to reproduce joint function, constructing a safe and comfortable new knee to improve mobility and life quality.
- How can I contribute to my recovery when I am resting?
Wait for your physician’s authorization to turn on your side, stand up, sit off the bed and walk. You will need the help of a professional (nurse, nurse technician or physical therapist) whenever you need to change positions.
CAUTION: Do not place pillows or supports under the operated knee. Always keep the knee stretched.
- Once the physician authorizes it, how should I proceed?
- Turning over: place a thick pillow between your legs and bend your legs, turn in full, that is, turn your entire body at once (hips and chest) toward the NON-operated side.
- Sitting up and standing up: when you sit up, keep the operated knee stretched and use your other leg and arms for support.
- Showering: once your physician authorizes it, you may take a shower. Be very careful not to fall, since the wet floor and soap residues make the floors very slippery. It is safer and more comfortable to shower sitting down. You may use a shower chair or a sturdy plastic chair. In the first weeks, you should have the help of a family member.
- Which exercises can I do on my own? Do 2 series of 10 repetitions, twice a day:
To avoid thrombosis: move your feet back and forth as if you were stepping on the gas pedal of a car.
- Flex your leg and thigh muscles as if pressing your knee against the bed and hold it for 10 seconds.
- Open and close your legs (laterally, with your knees stretched).
- Lift and lower your leg with your knee stretched.
- Stretch the back part of your legs with the help of a strap/sheet and towel.
- Bend and stretch your knee (sitting on the edge of the bed or chair) up to 90°.
What should I prepare before I am discharged?
- Toilet seat: there is a proper toilet seat that can be adapted to the toilet to elevate it.
- You can also use a shower chair.
- A chair with a firm seat and arm rests.
- A walker or crutches (elbow or axillary) depending on the orientations given by your physician or physical therapist.
How can I get in and out of the car?
- Always sit on the front seat (passenger).
- The seat must be pushed back and reclined.
- After you are discharged, you will be taken to the car by the nursing team and guided as to the correct position to seat in.
Can I go up and down stairs?
- You should avoid stairs. Wait for your physician’s permission and follow your physical therapist’s recommendations.
- Going upstairs: put the non-operated leg on the step above and then bring up the operated leg with the crutches.
- Going downstairs: put the operated leg with the crutches on the step below and then bring down the non-operated leg.
- The crutches should accompany the operated leg going up and down the stairs.
Guidelines for home:
Walking: [ ] Walker__[ ] Elbow crutches__[ ] Axillary crutches
Climbing stairs:
[ ] With axillary crutches__[ ] With a handrail__[ ] As practiced in the hospital
Use of a knee immobilizer: [ ] No__[ ] Yes
Ice pack: [ ] No__[ ] Yes – 4 times/day, for 30 minutes
continue...
...Continuation **Annex I.** Postoperative orientations given to patients (example for total knee prosthesis)
**Physical therapy orientations for total knee arthroplasty**
Important guides to increase safety:
- Always turn on the lights before standing up. Never walk in the dark.
- Remove all rugs to avoid slipping.
- Be careful not to step on areas with indents, wires or objects.
- Avoid lifting heavy objects.
- Do not wear high heels. Choose low heels with non-slip soles.
- Use a shower chair to shower during the first weeks.
- Use a shower sponge with a long handle.
- Drive only after authorized by your physician.
- Resume sexual activities only after authorized by your physican and respect the limitations from your surgery.
Contact your doctor in the following situations:
- Intense pain in the operated leg.
- If the operated leg is very swollen, red and hot.
- Yellowish discharge on the surgical incision.
- Difficulty breathing.
- Fever.
Other orientations: I state that I have received the orientations described above and the first copy of this form.
Full name:
Signature:
Hospital Physical Therapy Division – phone: (xxx) xxx-xxxx

## Annex II

**Table:**

Demonstration and training in the Practical Life Room
**Items addressed**
Stairs – going up
- Go up with the dominant, non-operated, non-injured leg.
- Use the handrail.
- If there is no handrail, have someone next to you.
- Wear non-slip, firm, closed-in shoes.
Ramp – going down
- Prefer stairs.
- Be careful with slippery floors.
- If there is no handrail, have someone next to you.
- Wear non-slip, firm, closed-in shoes.
Ramp – going up
- Prefer stairs.
- Be careful with slippery floors.
- If there is no handrail, have someone next to you.
- Wear non-slip, firm, closed-in shoes.
Stairs – going down
- Go down with the non-dominant, operated, injured leg.
- Use the handrail.
- If there is no handrail, have someone next to you.
- Wear non-slip, firm, closed-in shoes.
Bed
- Lie down according to the surgery.
- Turn according to the surgery.
- Positions permitted according to the surgery.
- Handle objects on your nightstand by turning according to the surgery.
continue...
...Continuation **Annex II.** Demonstration and training in the Practical Life Room
**Items addressed**
Spine
- In a block, flexing your lower limbs.
Hip
- With a hip abduction pillow or a large pillow between the lower limbs.
Knee
- With a pillow between the knees.
Rugs
- Remove or secure all rugs.
Office
- Adjust the angle of the seat.
- Adjust the height of the screen.
- Handling objects on the desk.
Spine
- Do not twist the body
Hip
- Do not flex the hip more than 90°.
Knee
- No limitations.
Kitchen
- Height of the counter.
- Footrest.
- Reaching objects in lower drawers, oven and high cabinets.
- How to carry shopping bags.
Laundry room
- Avoid rotating the body to reach objects that are far away.
- Bending over to pick up boxes from the floor.
Closet
- Long clothing, shoes with laces.
- Using a shoehorn with a long handle.
Bathroom
- Brushing teeth.
- Using a plastic chair or stool in the shower.
- Washing and drying feet while seating.
- For hip prostheses, using a sponge with a long handle.
- Using a non-slip mat.
- Using the toilet (for the hip, use a device to elevate the seat).
- Avoid rotating to reach for the toilet paper.
- Avoid rotating to throw the paper in the trash can.
- Use trash cans with a pedal.
