# Supplementary material for: Impact of demonstration in a realistic simulation environment as a postoperative education in patients’ experience
Source: Einstein (Sao Paulo). 2020 Mar 13;18:eAO4831. doi: 10.31744/einstein_journal/2020AO4831 (PMC7069733; doi:10.31744/einstein_journal/2020AO4831)
Supplement: Translation

# Translation: Impacto do uso de técnicas de demonstração em ambiente de simulação realística como forma de educação pós-operatória na experiência do paciente internado

**Authors:** Luciana Gardin Barbosa, Cinthya da Silva Frazão

**Affiliations:** 1 Brasil Associação de Assistência à Criança Deficiente, São Paulo, SP, Brasil

**Keywords:** Educação em saúde, Procedimentos ortopédicos, Satisfação do paciente, Treinamento por simulação

## Abstract

**Objetivo:**

Avaliar o impacto do treinamento no Ambiente Vida Prática na experiência do paciente durante a internação.

**Métodos:**

Pacientes em pós-operatório de cirurgias ortopédicas foram randomizados em dois grupos (Controle e Intervenção). O Grupo Controle recebeu orientações por escrito quanto ao pós-operatório, e o Grupo Intervenção recebeu adicionalmente uma sessão de demonstração e treinamento em um ambiente criado para simular uma casa e seus cômodos (sala, quarto, cozinha, lavanderia e banheiro) com profissional fisioterapeuta. Os participantes de ambos os grupos responderam o Questionário de Avaliação do Paciente Internado Relativo aos Sistemas e Prestadores de Cuidados de Saúde no dia da alta hospitalar.

**Resultados:**

Foram analisados 68 indivíduos, sendo 30 (44,1%) do Grupo Controle e 38 (55,9%) do Grupo Intervenção. O escore do Questionário de Avaliação do Paciente Internado Relativo aos Sistemas e Prestadores de Cuidados de Saúde foi semelhante entre os dois grupos (p=0,496).

**Conclusão:**

Não houve influência da intervenção proposta nos resultados do Questionário de Avaliação do Paciente Internado Relativo aos Sistemas e Prestadores de Cuidados de Saúde, talvez por limitação do instrumento ou por sua aplicação com o paciente ainda internado. Entretanto, por relatos dos pacientes do Grupo Intervenção sobre maior preparo e segurança para a execução das atividades do cotidiano, acredita-se que abordagens de educação do paciente por meio de demonstração devam ser inseridas como parte do processo de preparação para a alta, sempre que possível.

## INTRODUÇÃO

Cirurgias ortopédicas de grande porte, como correção de escoliose, hérnias de disco e artroplastias de quadril ou joelho, podem trazer limitações na execução de atividades da vida diária, como tomar banho, vestir-se, caminhar ou preparar as refeições no momento do retorno para casa.^( 1 )^Estudos sugerem que grandes procedimentos cirúrgicos podem resultar em condição de cinesiofobia temporária do paciente, por medo da execução de movimentos ou tarefas que possam vir a prejudicar o resultado cirúrgico.^( 2 , 3 )^Durante os primeiros dias após a cirurgia, pode haver perda total ou parcial da independência, sobretudo em pacientes idosos.^( 4 - 6 )^

A literatura recomenda que as condições para alta hospitalar devam incluir, além do controle da dor, adequadas amplitudes de movimento, força muscular e condições mínimas em realizar transferências e deambular, ou seja, que suas habilidades pré-operatórias permaneçam inalteradas ou minimamente reduzidas após a cirurgia.^( 7 , 8 )^Programas de reabilitação na fase intra-hospitalar que incluam educação do paciente por meio de orientações verbais ou escritas, demonstração ou treinamento de atividades diárias podem resultar em recuperações mais rápidas e, inclusive, em redução dos custos com reinternações, por complicações relacionadas ao imobilismo ou ocasião de acidentes domésticos, como queda, por exemplo.^( 5 )^

Equipes assistenciais utilizam impressos como forma de educação dos pacientes quanto às atividades permitidas, as que inspiram cuidados específicos ou as que devem ser evitadas após a alta para casa, de acordo com o tratamento realizado durante a internação. Ações que proporcionam a antecipação das dificuldades de forma prática e com acompanhamento tendem a proporcionar maior segurança durante o período pós-operatório no retorno ao lar.^( 5 )^

O Questionário de Avaliação do Paciente Internado Relativo aos Sistemas e Prestadores de Cuidados de Saúde (HCAHPS - *Hospital Consumer Assessment of Healthcare Providers and Systems* ),^( 9 )^que se trata de um questionário validado que mensura a satisfação do paciente com os serviços prestados durante a internação, tem sido utilizado como instrumento de avaliação dos pacientes e familiares quanto aos serviços prestados durante o período de internação. Ele contém 32 questões, divididas em domínios, sendo eles comunicação do médico (respeito, habilidade de ouvir as demandas dos pacientes e capacidade de comunicação dos médicos); comunicação do enfermeiro (respeito, habilidade de ouvir as demandas dos pacientes e capacidade de comunicação dos enfermeiros); receptividade da equipe (resposta aos sinais de chamada e às necessidades de ida ao banheiro); ambiente hospitalar (limpeza e sossego do hospital); gestão da dor (capacidade da equipe em diminuir a dor física dos pacientes); comunicação sobre medicação (explicação sobre medicamentos para os pacientes); informação de saída (preparo do paciente para sair do hospital); serviços de alimentação (qualidade dos alimentos e da cortesia daqueles que servem); avaliação geral do hospital (avaliação hospitalar em uma escala de 1 a 10).

Em dezembro de 2017, foi inaugurado, no hospital da Associação de Assistência à Criança Deficiente (AACD), o Ambiente Vida Prática, que simula os cômodos de uma casa. Neste espaço, o paciente pode contar com auxílio de um profissional de fisioterapia que o orienta, demonstra e dá a oportunidade ao paciente para simular atividades diárias, como deitar-se e levantar-se de uma cama, usar o vaso sanitário, tomar banho, usar o computador em uma mesa de trabalho ou até mesmo preparar uma refeição rápida. O objetivo é antecipar as dificuldades que podem ser encontradas após a alta hospitalar, explicar como superá-las e manter-se ativo dentro dos limites de segurança para sua recuperação. Os pacientes são levados a esse ambiente tão logo sua condição clínica permita, ou seja, quando não referem dor incapacitante, apresentam sintomas ou mal-estar de qualquer natureza.

Dessa forma, acredita-se que, além das explicações e cartilhas informativas quanto ao pós-operatório, a oportunidade de simulação das atividades cotidianas pode contribuir para a melhor recuperação do paciente e reduzir o risco de complicações.

## OBJETIVO

Avaliar o impacto do treinamento no Ambiente Vida Prática para paciente internados.

## MÉTODOS

Foram incluídos todos os pacientes internados entre junho e agosto de 2018, em pós-operatório de cirurgias ortopédicas de hérnias de disco cervicais, hérnias de disco lombares, correção de escoliose idiopática e artroplastias totais de quadril e joelho. Os sujeitos que apresentavam alterações cognitivas, clínicas ou motoras limitantes para participar da intervenção foram excluídos do estudo.

Inicialmente, os leitos do hospital foram randomizados por meio de um programa de randomização *online* em dois grupos (Controle e Intervenção).^( 10 )^A partir daí, os pacientes foram abordados conforme o leito ao qual foram alocados. O Grupo Controle recebeu orientações impressas já utilizadas no serviço (Anexo 1), e o Intervenção recebeu, além da orientação impressa, uma sessão de demonstração e treinamento no Ambiente Vida Prática (Anexo 2), com foco em prevenção de riscos e orientações para manutenção da funcionalidade no período pós-operatório. As figuras 1 a 3 mostram o Ambiente Vida Prática e seus cômodos.

**Figura 1:** Ambiente Vida Prática

**Figura 2:** Treinamento e demonstração ao paciente no Ambiente Vida Prática (lavanderia, escritório, cozinha e quarto)

**Figura 3:** Treinamento e demonstração ao paciente no Ambiente Vida Prática (rampas, escadas, vaso sanitário e banho)

O questionário HCAHPS foi entregue aos pacientes incluídos no estudo no dia da alta hospitalar. Os indivíduos o preencheram e devolveram a um pesquisador cego à randomização. Aos pacientes do Grupo Controle, foi dada a oportunidade de passar pelo ambiente na primeira semana após a alta hospitalar.

Todas as variáveis foram avaliadas com estatística descritiva. As variáveis resposta, relativas ao escore do HCAHPS, foram testadas de duas formas: HCAHPS geral, com todas as respostas obtidas, e HCAHPS alta, com apenas as questões voltadas à preparação para alta do paciente (questões 18, 19 e 20), para verificar a aderência à distribuição normal. Posteriormente, foi utilizado o teste de Mann-Whitney e o teste Kruskal-Wallis para comparação dos escores entre grupos, sexos e diagnósticos. As variáveis quantitativas foram comparadas entre grupos com o teste do χ^2^. Os intervalos de confiança das frequências foram obtidos por meio de *bootstrapping* .

Trata-se de um ensaio clínico randomizado, controlado e cego, aprovado pelo Comitê de Ética em Pesquisa do Hospital e Centro de Reabilitação da Associação de Assistência à Criança Deficiente (CAAE: 88131418.0.0000.0085), com parecer 2.792.735.

## RESULTADOS

Dos 71 sujeitos que iniciaram e pesquisa, 3 foram excluídos por não terem respondido o questionário integralmente (1 do Grupo Controle e 2 do Grupo Intervenção). Dos 68 indivíduos analisados, 30 (44,1%) eram do Grupo Controle e 38 (55,9%) do Grupo Intervenção. A variável HCAHPS geral apresentou distribuição normal e o escore HCAHPS alta, não (p<0,001); por isso, optou-se por utilizar testes não paramétricos.

A variável sexo apresentou diferença entre os grupos (p=0,027). No Grupo Intervenção, 23,7% eram do sexo masculino *versus* 50% no Controle. Observou-se distribuição homogênea dos diagnósticos entre os grupos (p=0,838) e entre os sexos (p=0,316). Os resultados da estatística descritiva das variáveis qualitativas, separadas por grupo, estão expostos na tabela 1 .

**Tabela 1:** Variáveis qualitativas do estudo para os Grupos Controle e Intervenção

	Grupo Controle		Grupo Intervenção	
	n (%)	IC95%	n (%)	IC95%
Sexo				
Feminino	15 (50,0)	32,4-66,7	29 (76,3)	62,9-88,2
Masculino	15 (50,0)	32,4-66,7	9 (23,7)	11,8-37,1
Diagnóstico				
Escoliose Idiopática	3 (10,0)	0,0-22,6	8 (21,1)	8,8-35,3
Hérnia de disco cervical	7 (23,3)	9,7-40,0	9 (23,7)	11,1-38,1
Hérnia de disco lombar	14 (46,7)	29,0-64,0	16 (42,1)	26,5-57,9
Artroplastia de joelho	2 (6,7)	0,0-17,4	1 (2,6)	0,0-9,1
Artroplastia de quadril	3 (10,0)	0,0-22,6	3 (7,9)	0,0-17,5
Prótese de joelho	1 (3,3)	0,0-11,1	1 (2,6)	0,0-9,1

IC95%: intervalo de confiança 95%.

As variáveis quantitativas são apresentadas na tabela 2 , na qual observamos que os valores de média e mediana foram semelhantes.

**Tabela 2:** Variáveis quantitativas do estudo para os Grupos Controle e Intervenção

	Grupo Controle		Grupo Intervenção	
	Média±DP (mediana)	P25-P75	Média±DP (mediana)	P25-P75
Idade	46,1±13,6 (46,5)	39,0-53	43,8±18,9 (44,5)	37-52
IMC	28,8±6,0 (27,9)	25,6-31,9	29,3±18,7 (27,1)	22,4-29,7
Escore HCAHPS geral	79,5±6,9 (79)	75-86	80,8±6,0 (81)	76-86
Escore HCAHPS alta	3,6±0,9 (3)	3-4	3,5±1,0 (3)	3-4

DP: desvio padrão; IMC: índice de massa corporal; HCAHPS: Avaliação do Paciente Internado Relativo aos Sistemas e Prestadores de Cuidados de Saúde.

Ao avaliar as variáveis escore HCAHPS geral e alta, a distribuição foi semelhante entre os Grupos Intervenção e Controle (p=0,496 e p=0,400, respectivamente). Visualmente, os gráficos de *boxplot* ( Figura 4 ) mostram a sobreposição dos dois grupos, em concordância com o resultado do teste estatístico.

**Figura 4:** *Boxplot* para comparação dos grupos em relação ao escore Avaliação do Paciente Internado Relativo aos Sistemas e Prestadores de Cuidados de Saúde geral e alta HCAHPS: Avaliação do Paciente Internado Relativo aos Sistemas e Prestadores de Cuidados de Saúde.

A comparação das questões individuais do HCAHPS entre os grupos mostrou que apenas na questão 11 houve diferença entre os grupos (p=0,044); as demais não mostraram significância estatística (entre p=0,116 e p=1,0).

Não houve diferença nas avaliações HCAHPS geral, alta, ou em questões individuais (significância entre p=0,119 e p=1,0) entre os sexos.

Houve diferença na avaliação HCAHPS alta entre os diagnósticos. Os pacientes com diagnóstico de artroplastia total de quadril apresentaram escore superior àqueles com diagnóstico de artroplastia total de joelho, hérnia de disco cervical e hérnia de disco lombar (p=0,024, p=0,027 e p=0,005, respectivamente). Porém, não houve diferença entre os Grupos Controle e Intervenção dentro de cada diagnóstico. Esta diferença no escore HCAHPS alta se deveu a um melhor desempenho dos pacientes com diagnóstico de artroscopia total de quadril na questão 19 (p=0,001).

## DISCUSSÃO

Pacientes com patologias degenerativas do sistema osteomuscular vivenciam limitações diárias devido a sintomas como dor, limitação articular e disfunção do movimento, o que pode acarretar em redução das atividades física e social.^( 11 - 13 )^Page et al., citam ainda impacto no sono e sofrimento emocional dentre as limitações referidas por pacientes portadores de patologias degenerativas de ombro.^( 14 )^

As cirurgias têm como objetivo principal a redução dessas limitações, e a reabilitação deve proporcionar a recuperação das funções motora e respiratória o mais brevemente possível. A participação do paciente nesse processo é determinante para melhores desfechos.

McCormick et al., afirmam que existe uma tendência a realizarmos avaliações equivocadas das necessidades dos pacientes quando utilizamos métodos tradicionais, baseados apenas na experiência dos profissionais de saúde, pois tais métodos podem não descrever a percepção do paciente sobre seu estado de saúde.^( 15 )^

Quando o cuidado é centrado no paciente e na família, respeitando suas preferências e crenças, adaptando-se às suas necessidades e viabilizando a continuidade do cuidado, os resultados e a experiências durante a hospitalização são superiores, contribuindo para melhora na saúde mental e diminuição do tempo de internação.^( 16 )^Goldfarb et al., ressaltam também que esse tipo de abordagem oferece cuidados preventivos, reduzindo o número de tratamentos indesejados e, consequentemente, aprimorando a qualidade do atendimento, com relatos de satisfação com os serviços prestados.^( 17 )^

Os cuidados com o paciente na transição da hospitalização até a alta para casa devem ser personalizados, priorizando a qualidade, para mitigar riscos no período pós-alta hospitalar, que podem ocorrer caso não haja um processo adequado de orientação e educação do indivíduo ainda na fase hospitalar. Estes mecanismos estão diretamente associados a uma melhor adesão do paciente ao tratamento, tanto nas populações pediátricas, como nas adultas e idosas. As orientações escritas geralmente são pouco explicativas e genéricas.^( 18 )^

Na população de nosso estudo, a cirurgia de maior prevalência foi a de hérnia de disco lombar, por tratar-se de um serviço de referência em cirurgia de coluna. Também foram predominantes pacientes de meia-idade, obesos e do sexo feminino. Apesar de haver diferença de frequência de sexos entre os grupos, isso não implicou no resultado, não havendo diferença no escore final em relação ao sexo.

Os resultados não mostram diferença significativa entre os Grupos Intervenção e Controle em relação aos escores do HCAHPS geral e HCAHPS alta. Este resultado pode ser consequência de um número de pacientes insuficiente para demostrar o efeito da intervenção ou de uma ferramenta de mensuração inadequada.

Apesar de não ter sido mensurado de forma objetiva, vale ressaltar que, durante a prática clínica, grande parte dos familiares e cuidadores demonstravam não estar seguros e adequadamente preparados para cuidar do paciente pós-alta hospitalar. Após as intervenções, seja com as orientações por escrito ou pela sessão prática no Ambiente Vida Prática, os relatos eram de maior segurança e satisfação com os cuidados prestados, o que corrobora a importância da estruturação desse processo nas instituições de saúde.

Recente estudo relata que a utilização de recursos visuais estabelece maior envolvimento e melhor compreensão dos pacientes durante as instruções para alta.^( 18 )^Porém, nosso estudo não foi conclusivo para avaliar o efeito dos recursos instituídos no hospital da AACD na experiência do paciente e sugerimos que mais pesquisas sejam realizadas.

O momento da aplicação do questionário também pode ter sido uma importante limitação do estudo, já que o preconizado pela metodologia do HCAHPS é que ele seja respondido após alguns dias em casa. Devido a limitações internas à nossa equipe de pesquisa, o questionário precisou ser aplicado no momento da alta do paciente, e acredita-se que a percepção do benefício da intervenção aplicada não ainda seja notável nessa fase.

Um achado do estudo foi a diferença na avaliação HCAHPS alta entre os diagnósticos. Os pacientes com diagnóstico de artroplastia total de quadril apresentaram escore superior àqueles com diagnóstico de artroplastia total de joelho, hérnia de disco cervical e hérnia de disco lombar. A diferença foi relacionada à pontuação da questão 19, que considera se “durante esta hospitalização, os médicos, pessoal de enfermagem, ou outro pessoal do hospital, falaram-lhe sobre se teria a ajuda necessária após ter alta do hospital”, e as respostas “não” ocorreram principalmente no grupo de pacientes submetidos à artroplastia de quadril. Isso pode denotar dificuldade em dimensionar a necessidade de ajuda no período pós-operatório para essa população específica e, apesar desse resultado não ter sido objeto desse estudo, foi trazida informação importante, que será considerada na construção de nossos processos de educação dos pacientes.

Assim, ainda que os resultados obtidos não expressem diferenças significantes e não sejam correspondentes a maior satisfação referida pelos pacientes, acredita-se que quanto mais abordagens diferentes o hospital puder implementar no processo de educação do paciente e familiares durante a internação, tão melhor será a continuidade do cuidado prestado em domicílio. Talvez isso possa trazer algum impacto positivo em relação à recuperação pós-operatória em si. Entretanto, para mensurar este efeito, um estudo específico precisaria ser realizado.

## CONCLUSÃO

Nosso estudo não foi capaz de demonstrar influência do treinamento no Ambiente Vida Prática na satisfação do paciente com os serviços prestados durante a internação, mensurada por meio do Questionário de Avaliação do Paciente Internado Relativo aos Sistemas e Prestadores de Cuidados de Saúde no momento da alta hospitalar. Um estudo adicional seria necessário, com aplicação do instrumento na primeira semana após a alta, ou de uma ferramenta mais sensível à intervenção. Entretanto, relatos dos pacientes que sofreram a intervenção mostraram maior preparo e segurança para a execução das atividades do cotidiano. Acredita-se que abordagens de educação do paciente por meio de demonstração devam ser inseridas como parte do processo de preparação para a alta, sempre que possível.

## Anexo I

**Table:**

Orientações pós-operatórias fornecidas aos pacientes (exemplo para prótese total de joelho)
**Orientações fisioterapêuticas para a cirurgia de artroplastia total de joelho**
Este manual foi elaborado pela equipe de fisioterapia do hospital com a finalidade orientar os cuidados no pós-operatório.
Seguindo as orientações a seguir, você estará contribuindo para o sucesso da cirurgia e poderá retornar mais brevemente às suas atividades diárias, resgatando sua independência.
Abaixo seguem algumas orientações dadas a você, durante a internação, que devem ser seguidas em casa até o retorno ao seu médico:
Definição
Artroplastia total de joelho é a substituição da articulação original com problemas por uma nova articulação denominada prótese de joelho. É feita por materiais especiais que tentam reproduzir a função articular, compondo um novo joelho seguro e confortável, com o objetivo de melhorar a mobilidade e a qualidade de vida.
- Como posso colaborar com minha recuperação quando estiver em repouso?
Aguarde a autorização do seu médico para virar de lado, levantar, sentar fora do leito e andar. Você precisará da ajuda de um profissional (enfermeiro, técnico de enfermagem ou fisioterapeuta, sempre que necessitar mudar de posição).
ATENÇÃO: Não coloque almofadas ou apoios embaixo do joelho operado. Mantenha sempre o joelho esticado.
- Quando autorizado, como devo proceder?
- Virar de lado: coloque um travesseiro espesso entre as pernas e dobre suas pernas, vire em bloco, ou seja, todo o corpo de uma vez (quadril e tronco), para o lado NÃO operado.
- Sentar e levantar: ao levantar e sentar mantenha o joelho operado esticado e faça o apoio na outra perna e nos braços.
- Tomar banho: após liberação médica, o banho poderá ser realizado no chuveiro. Deve-se tomar muito cuidado para evitar quedas, já que o banheiro molhado e com resíduos de sabonete torna-se muito escorregadio. É mais seguro e confortável tomar banho sentado. Pode-se utilizar uma cadeira higiênica ou uma cadeira de plástico firme. Nas primeiras semanas, você deverá contar com a ajuda de algum familiar.
- Quais exercícios posso fazer sozinho? Realizar 2 séries de 10 repetições, 2 vezes/dia:
Para prevenir trombose: movimente seus pés para frente e para trás, como se estivesse acelerando e desacelerando um carro.
- Contrair os músculos da perna e da coxa como se estivesse apertando o joelho contra a cama e segure por 10 segundos.
- Abrir e fechar a perna (lateral com o joelho esticado).
- Subir e abaixar a perna com o joelho esticado.
- Alongar parte posterior da perna com auxílio de faixa/lençol e toalha.
- Dobrar e esticar o joelho (sentado na beira da cama ou cadeira) até 90°.
O que devo providenciar antes de receber alta?
- Assento de vaso sanitário: existe um assento apropriado para ser adaptado ao vaso sanitário, o qual eleva a altura do vaso.
- Existe também a opção de utilizar a cadeira higiênica.
- Cadeira com assento firme e apoio para braços.
- Andador ou muletas (canadenses ou axilares), de acordo com a orientação do fisioterapeuta e/ou do seu médico.
Como farei para entrar e sair do carro?
- Sente-se sempre no banco da frente (do passageiro).
- O banco deverá estar com o encosto reclinado e afastado para trás.
- Após a liberação da alta, você será encaminhado até o carro pela equipe de enfermagem e será orientado sobre o posicionamento correto para se sentar.
Posso subir e descer escadas?
- Você deve evitar escadas. Aguarde a liberação de seu médico e siga a orientação do fisioterapeuta.
- Para subir: coloque a perna não operada no degrau superior, em seguida, leve a perna operada juntamente das muletas.
- Para descer: coloque a perna operada juntamente das muletas no degrau inferior, em seguida leve a perna não operada.
- As muletas devem acompanhar a perna operada, tanto na subida como na descida.
Cuidados e orientações para casa:
Andar: [ ] Andador__[ ] Muleta canadense__[ ] Muleta axilar
Subir escadas:
[ ] Com auxílio de muleta axilar__[ ] Com apoio em corrimão__[ ] Conforme treinado durante a internação
Uso de imobilizador de joelho: [ ] Não__[ ] Sim
Aplicação de gelo: [ ] Não__[ ] Sim – 4 vezes/dia, por 30 minutos
continua...
...Continuação **Anexo I.** Orientações pós-operatórias fornecidas aos pacientes (exemplo para prótese total de joelho)
**Orientações fisioterapêuticas para a cirurgia de artroplastia total de joelho**
Orientações importantes para aumentar sua segurança:
- Acenda sempre a luz antes de se levantar. Nunca ande no escuro.
- Retire os tapetes para não correr o risco de escorregar;
- Tome cuidado para não pisar em locais com buracos, fios ou objetos.
- Evite carregar peso.
- Não use calçado com salto alto. Prefira salto baixo e com solado antiderrapante.
- Utilize cadeira higiênica própria para banho nas primeiras semanas.
- Utilize bucha de banho com prolongador.
- Dirija carro apenas após a liberação do seu médico.
- Retorne à vida sexual após a liberação do médico, respeitando as restrições da cirurgia.
Procure seu médico nas seguintes situações:
- Dor intensa na perna operada.
- Perna operada muito inchada, vermelha e quente.
- Saída de secreção amarelada pela incisão cirúrgica.
- Dificuldade de respirar.
- Febre.
Outras orientações: afirmo que recebi as orientações acima descritas, bem como a primeira via desse formulário.
Nome completo:
Assinatura:
Setor de Fisioterapia Hospitalar – telefone: (xx) xxxx-xxxx

## Anexo II

**Table:**

Demonstração e treinamento no ambiente vida prática
**Itens abordados**
Escada – subir
- Subir com a perna dominante, não operada ou sem *deficit* .
- Usar o corrimão.
- Se não houver corrimão, com alguém ao lado.
- Usar calçado antiderrapante, fechado e firme.
Rampa – descer
- Preferir escadas.
- Cuidado com pisos escorregadios.
- Se não houver corrimão, com alguém ao lado.
- Usar calçado antiderrapante, fechado e firme.
Rampa – subir
- Preferir escadas.
- Cuidado com pisos escorregadios.
- Se não houver corrimão, com alguém ao lado.
- Usar calçado antiderrapante, fechado e firme.
Escada – descer
- Descer com a perna não dominante, operada ou com *deficit* .
- Usar o corrimão.
- Se não houver corrimão, com alguém ao lado.
- Usar calçado antiderrapante, fechado e firme.
Cama
- Deitar-se de acordo com a cirurgia.
- Virar-se de acordo com a cirurgia.
- Posições permitidas de acordo com a cirurgia.
- Manipular objetos no criado-mudo virando-se de acordo com a cirurgia.
continua...
...Continuação **Anexo II.** Demonstração e treinamento no ambiente vida prática
**Itens abordados**
Coluna
- Em bloco, com membros inferiores flexionados.
Quadril
- Com triângulo de abdução ou travesseiro grande entre os membros inferiores.
Joelho
- Com travesseiro entre os joelhos.
Tapetes
- Eliminar tapetes ou prendê-los.
Escritório
- Ajuste da angulação do assento.
- Ajuste da altura da tela.
- Manipulação de objetos em cima da mesa de trabalho.
Coluna
- Sem torção de tronco
Quadril
- Sem flexão de quadril acima de 90°.
Joelho
- Sem limitações.
Cozinha
- Altura da bancada.
- Degrau para apoio dos pés.
- Alcance de objetos em gavetas baixas, forno e armários altos.
- Como carregar sacolas de compras.
Lavanderia
- Evitar rotação de tronco para alcance de objetos em locais distantes.
- Abaixar-se para pegar caixas do chão.
Armário
- Roupas longas, calçados com cadarço.
- Uso de calçadeira com cabo longo.
Banheiro
- Escovar os dentes.
- Usar cadeira plástica ou banco plástico no chuveiro.
- Lavar e secar os pés sentado.
- Para próteses de quadril, usar buchas com cabo longo.
- Usar tapete antiderrapante.
- Uso do vaso sanitário (para quadril, usar o elevador).
- Evitar rotações de para pegar papel higiênico.
- Evitar rotações para jogar papel na lixeira.
- Usar lixeiras de pedal.
